# The double burden of malnutrition in a rural health and demographic surveillance system site in South Africa: a study of primary schoolchildren and their mothers

**DOI:** 10.1186/s12889-019-7412-y

**Published:** 2019-08-09

**Authors:** Perpetua Modjadji, Sphiwe Madiba

**Affiliations:** Department of Public Health, Sefako Makgatho Health Sciences University, School of Health Care Sciences, P O Box 215, Ga-Rankuwa, MEDUNSA, 0204 South Africa

**Keywords:** Thinness, Overweight/obesity, Schoolchildren, South Africa, Rural context

## Abstract

**Background:**

In South Africa, the occurrence of the double burden of malnutrition is on the rise at a household level predisposing children and their mothers to negative health outcomes. However, few studies have been conducted at a household level. Therefore, we studied a double burden of malnutrition using child-mother pairs in a rural setting.

**Methods:**

A cross-sectional quantitative survey was conducted among 508 child-mother pairs selected from primary schools using a multistage sampling in a rural Dikgale Health and Demographic Site in Limpopo Province, South Africa. Anthropometric measurements of children and mothers, and socio-demographic data were collected. WHO AnthroPlus was used to generate body-mass-index z-scores of children and the BMI was used to indicate overweight and obesity among the mothers. Mann Whitney test was used to compare the means of variables between sexes and age groups, while the prevalence of thinness and overweight/obesity were compared using a chi-square. Multivariate logistic regression with a stepwise backward elimination procedure, controlling for confounding, was used to determine the association between the thinness and overweight/obesity and the covariates.

**Results:**

Twenty five percent (25%) of the children were thin, 4% were overweight and 1% obese, while mothers were overweight (27.4%) and 42.3% obesity (42.3%) were observed among the mothers. The odds of being thin were higher in boys than in girls (AOR = 1.53, 95%CI: 1.01–2.35). Overweight/obese mothers were more likely to have thin children (AOR = 1.48, 95% CI: 1.01–2.18) and less likely to have overweight/obese children (AOR = 0.18, 95%CI: 0.07–0.46).

**Conclusion:**

A double burden of malnutrition was observed on a household level with thinness among children and overweight/obesity among mothers. A need to address the dual problems of undernutrition and rapidly rising trends of overweight/obesity cannot be over-emphasized.

## Background

The coexistence of undernutrition (i.e. thinness, stunting and underweight) and over-nutrition (i.e. overweight and obesity) or diet-related non-communicable diseases is known as the double burden of malnutrition [1]. The double burden of malnutrition was previously commonly observed in developed and affluent communities, but, as early as 1996, it was noticed in the low-to-middle income countries (LMICs) [2–4]. In LMICs, the double burden of malnutrition was initially documented in adults, but it has also been seen in children from Brazil, China, Russia and South Africa [2, 4–6]. The double burden of malnutrition is often assed by reporting the prevalence of undernutrition and the prevalence of a measure of over nutrition within households, specific communities, and regions or at a country level [4].

The double burden of malnutrition can manifest at the individual level when the development of two or more forms of malnutrition is observed simultaneously [4]. For example, an individual child often manifests as stunting or micronutrient deficiencies co-occurring with overweight or obesity. On the household level, the double burden of malnutrition may arise from contrasting forms of malnutrition in multiple family members. For example, when a mother is overweight and a child is underweight. While at the population level, both undernutrition and overweight are prevalent in the same community, nation, or region [1, 7]. Studies conducted in LMICs, such as Brazil, China and Russia reported high prevalence of underweight or stunting and overweight in the same population, or on household levels with both obese and stunted or underweight individuals [5, 6].

According to the UNICEF, in South Africa, 13% of children under 5 years are overweight while 27% are stunted. Among teenagers aged 15–19 years, overweight ranges from 8.6–27% while stunting is 12.9% [8]. In an earlier study conducted among children and adolescents aged 1–20 years, in a rural health and demographic surveillance site, the results showed the double burden of malnutrition through the presence of undernutrition (i.e. wasting, stunting and underweight) at an early age, with marked level of overweight/obesity in adolescents [3]. Similarly, another study conducted in the rural villages showed that children aged three years had the double burden of malnutrition. [9]. Furthermore, within the same region of the study, high prevalence of overweight and obesity was reported among women of childbearing age [10–14]. Whereas overweight/obesity was prevalent among women in urban settings, Popkin et al. [15], argue that the prevalence is now high among rural women in LMICs, suggesting that, rural women are quickly catching-up with their urban counterparts.

South Africa and other LMICs, are undergoing nutrition and lifestyle transitions, increasing the trend of over-nutrition at population level, despite persistent undernutrition among children [16]. The overall transition has created a nutrition transition linked with obesity and hunger because of the structure of prices and food availability [15]. The nutrition transition in South Africa is complex and is characterised by urban migration, economic, and social transitions [17]. While persistent undernutrition in South Africa is attributed to poverty and household food insecurity. The emerging overweight/obesity are attributed to the consumption of high energy-dense foods and little physical activity [18]. Similarly, Tzioumis and Adair [4] reported that the persistent pockets of undernutrition in LMICs is a result of the widening of economic disparities characterized by poverty and poor access to resources.

School age is the active growing phase of childhood and a dynamic period of physical growth and mental development [19]. The double burden of malnutrition adversely affects the growth, health, intellectual development, and school attendance of school-aged children [7, 20]. Through its effects on health, the double burden of malnutrition increases the costs of health care, reduces productivity, and slows economic growth, which in turn can perpetuate a cycle of poverty and ill-health [1, 21]. In South Africa, the occurrence of the double burden of malnutrition is observed among schoolchildren, despite the implemented National School Nutritional Programme (NSNP). The NSNP was introduced by the South African government, in 1994, to provide schoolchildren with nutritious meals. The Healthy Active Kids South Africa (HAKSA) suggests that the occurrence of double burden of malnutrition indicates that the NSNP is not meeting its objectives of providing optimal nutritious meals [22].

In South Africa, there are few studies that have been conducted on the double burden of malnutrition among children and adolescents in both the rural and the urban settings [3, 9, 23–25], but the urban-rural difference of the double burden of malnutrition that may exist has not been well documented [15]. Furthermore, not much has been done to investigate the presence of the double burden of malnutrition at a household level using child-mother pairs in South Africa. Therefore, we studied the double burden of malnutrition and determined the prevalence and determinants of thinness, overweight and obesity using child-mother pairs in a rural health and demographic surveillance system site, South Africa. The United Nations has indicated the importance of addressing the double burden of malnutrition in order to achieve the Sustainable Development Goal 2 of ending all forms of malnutrition by 2030 [26]. Although this site has been well researched [27], to our knowledge, there is no baseline data on malnutrition in schoolchildren.

## Methods

### Study design

This paper is part of a larger study, which determined the growth patterns of primary schoolchildren and the maternal factors influencing those growth patterns, an explored the influence of the cultural beliefs and practices of mothers on child growth and nutrition using a convergent mixed method. The study was conducted from August 2017 to December 2017. This paper reports on the cross sectional quantitative survey conducted to study double burden malnutrition and determined the prevalence and determinants of thinness, overweight and obesity using child-mother pairs.

### Study setting

The study was conducted in Dikgale Health and Demographic Surveillance System Site (DHDSSS). DHDSSS is a research-based rural site founded in 1995 and forms part of the International Network for the Demographic Evaluation of Populations and their Health (INDEPTH). INDEPTH is an umbrella organisation for a group of independent health research centres operating 43 Health and Demographic Surveillance Sites in 20 LMICs [28]. The site is situated approximately 40 km northeast of Polokwane, the capital city of Limpopo Province, in South Africa. The area constitutes communities made up of households clustered in 16 villages with a population of approximately 36,000. The area has poor infrastructure but electricity and mobile phone networks are found everywhere, while the piped water supply from taps is not continuous because of water interruption, hence, at most, residents access water which is delivered by the government water tanks [27]. A poor socioeconomic status characterised by high unemployment and poverty has been reported in this area [10, 11, 13].

There are 19 public primary schools across the villages of DHDSSS site, and the total enrolment number (EN) of learners was estimated at 7772 in 2016, ranging from an enrollment number of 112 children in the smallest school to 776 in the largest school [29]. The primary schools in this area belong to quintile three (Q3) schools, which receive the majority of their funding from the South African government [30].

### Participants

This was a child-mother paired study, and the study population comprised primary school learners and their mothers. The study excluded children who were younger than 5 years, or had physical disabilities that compromised their stature, or whose biological mothers were not available to participate.

### Sample size and sampling procedure

A multistage sampling technique was used to select schools. First, the schools were stratified by size of enrollment and five of the largest schools were selected. Within the selected schools, simple random sampling of grades, classes, and learners was carried out. The sample size was calculated using Rao software sample size calculator (Raosoft.com), recommended as one of the best practices for calculating sample sizes for surveys [31]. The software takes into consideration the population size, which was 7772, a 5% margin of error, a 95% confidence level, and a 50% response distribution. A minimum recommended sample was 367 learners. To cater for a non-response, the sample was increased by 30% to 477 learners. Each selected school was treated as a unit of analysis with a sample size of 93 learners increased by 10% to 103 per school to make a final sample was 515 learners. At data analysis, seven questionnaires had missing data above 10% and were excluded. The final sample was 508 child-mother pairs.

### Data collection

A structured interviewer-administered questionnaire translated from English to a local language (Sepedi) by the principal researcher who is fluent in the local language and English. The questionnaire took into consideration some determinants of nutritional status among children [32] and it covered a range of information on the socio-demographics and household situations of mothers, like the variables used in other studies conducted in the study area [10, 11, 27]. The current study used the tools that were previously translated into the local language of the study population. The study was piloted to test the feasibility of the study, particularly, the ability to recruit child-mother pairs in one of the primary schools that did not form part of the study sample situated at the outskirts of the study area. The study was feasible, there were no ambiguous questions, and therefore, the tool was not modified.

The principal researcher and four trained research assistants collected data from the participants. The weights and heights of the children and their mothers were measured using a well-calibrated, smart D-quip electronic scale and a height measuring board, respectively. Height was recorded to the nearest 0.1 cm and weight to the nearest 0.1 kg. All measurements were taken three times, and the average was taken. A non-stretchable plastic tape was used to measure mid-upper arm circumference (MUAC) among the children, and waist and hip circumferences of the mothers, and were recorded to nearest 0.1 cm. All measures were done according to WHO recommendations [33, 34] .

For the children, the anthropometric measurements were converted to BMI and BMI-for-age Z-scores (BMIZ) and compared to reference data for 5–19 years, using the WHO Anthro-plus computer programme [33]. The children were classified as thin if the BMIZ was less than or equal to -2SD. Overweight and obesity were taken as z-scores of +2SD and + 3SD, respectively. Z-scores above 1SD indicated a possible risk of overweight. The MUAC measurements were recorded at < 135 mm for mild acute malnutrition among children aged 5–9 years and < 160mmm for children aged 10–15 years [35]. For the mothers, the BMI was calculated as the weight in kilograms divided by the height in meters squared [BMI (kg/m^2^) = weight (kg)/height (m)^2^]. Normal BMI is within 19.0 to 24 kg/m^2^. Underweight was defined as BMI < 18.5 kg/m^2^, overweight as BMI: 25.0 to 29.9 kg/m^2^, and obesity as BMI ≥ 30.0 kg/m^2^. The cutoff point for central obesity among the mothers was a waist circumference ≥ 88 cm. The waist-hip ratio (WHR) was computed as the waist circumference divided by the hip circumference. The WHR cut-off point (i.e. abdominal obesity) for mothers was ≥0.85 [34].

### Statistical analysis

The data were analysed using STATA version 14. Descriptive statistics for the children’s age, body weight (W), height (H), BMI and MUAC were computed for the mean, the standard deviation (SD), the median and the interquartile range (IQR). The skewness and kurtosis test for normality was used to determine the distribution of variables. The variables with skewed distribution are presented as median (IQR). Comparisons of the means were done using the Mann-Whitney test, while the percentages of children with variables below, on and above the cut-off points were compared using a chi-square test. Bivariate and multivariate logistic regression analysis was used to determine the association between the nutritional status indicators of children, their thinness, overweight and obesity, and independent variables, which included child and maternal characteristics. Bivariate analyses were used to identify the association between the dependent variables and each of the independent variables. Independent variables that had an association at a *p*-value of 0.1 were used in multivariate logistic regression with a stepwise backward elimination procedure controlling for confounding. Adjusted odds ratios (AOR) with a 95% confidence interval (CI) were generated and used to determine the independent strength of the associations, and significance was considered at *P* < 0.05.

## Results

### Distribution of schoolchildren

A representative sample of 508 schoolchildren was obtained from the five largest primary schools in DHDSSS. Of the 508 participants, 209 (41%) were boys and 299 (59%) were girls. The study showed that the age range of children in primary schools of DHDSSS was 6–15 years, with mean age of 9.6 (SD = ± 2.2). Children were divided into two age groups; usually used in nutritional assessment of children [36, 37], with the consideration that schooling starts at 6 years in South Africa, which are 6–9 years (*n* = 254), with mean age of 7.7 years (SD = ±1.0), and 10–15 years (n = 254), with a mean age of 11.5 years (SD = ±1.2). The results showed that there were children who were aged 13–15 years in the primary schools of DHDSSS. The children who participated in this study were in learning grades 1 to 7.

### Nutritional indicators of children

In Table 1, the means of the ages and nutritional indicators of the children are compared by sex using the Mann-Whitney test. No significant differences of means for age, weight, height, MUAC, BMI and BMIZ were observed between the boys and the girls.

**Table 1:** Comparison of children’s variables by sex categories

Variables	All N = 508 Median(IQR)	Boys *N* = 209 Median(IQR)	Girls *N* = 299 Median(IQR)	P-Value
Age (years)	9.6 (8; 11)	9.6 (8; 11)	9.6 (8; 12)	0.77
Weight (kg)	31.1 (22.7; 36)	30.1 (22.7; 33.3)	31.7 (22.7; 39.2)	0.29
Height (cm)	135.5 (124; 145.2)	134.7 (124; 143.5)	136.1 (125.5; 147)	0.22
MUAC (cm)	176.5 (155; 190)	176.6 (155; 190)	176.5 (155; 190)	0.78
BMI (kg/cm^2^)	16.5 (14.6; 17.6)	16.2 (14.6; 17.1)	16.7 (14.5; 17.8)	0.24
BMIZ	−0.4 (−1.0; 0.4)	−0.4 (−1.1; − 0.4)	0.3 (− 0.9; 0.5)	0.23

Table 2 shows the means for the nutritional indicators of children compared by age groups (6–9 years and 10–15 years). Significant differences of means between the two age groups were observed for weight (*p* ≤ 0.0001), height (p ≤ 0.0001), and BMI (p ≤ 0.0001), but not for MUAC (*p* = 0.57) or BMIZ (*p* = 0.47).

**Table 2:** Comparison of children’s variables by age categories

Variables	All *N* = 508 Median(IQR)	6 - 9 years *N* = 254 Median(IQR)	10 - 15 years *N* = 254 Median(IQR)	P-Value
Age (years)	9.6 (8; 11)	7.7 (7; 9)	11.5 (10; 13)	≤ 0.0001*
Weight (kg)	31.1 (22.7; 36)	24.8 (20.6; 26.8)	37.3 (30.1; 43.8)	≤ 0.0001*
Height (cm)	135.5 (124; 145,2)	125.9 (119; 131)	145,1(137.4; 152)	≤ 0.0001*
MUAC (cm)	176.5 (155;190)	176.9 (155; 190)	176.5 (155; 190)	0.57
BMI (kg/cm^2^)	16.5 (14.6; 17.6)	15.5 (14.2; 16;2)	17.5 (15.4; 18,7)	≤0.0001*
BMIZ	−0.4 (− 1.0; 0.4)	−0.4 (− 1.0; 0.3)	−0.4 (− 1.0; 0.5)	0.47

* indicates significance

**Fig. 1:**
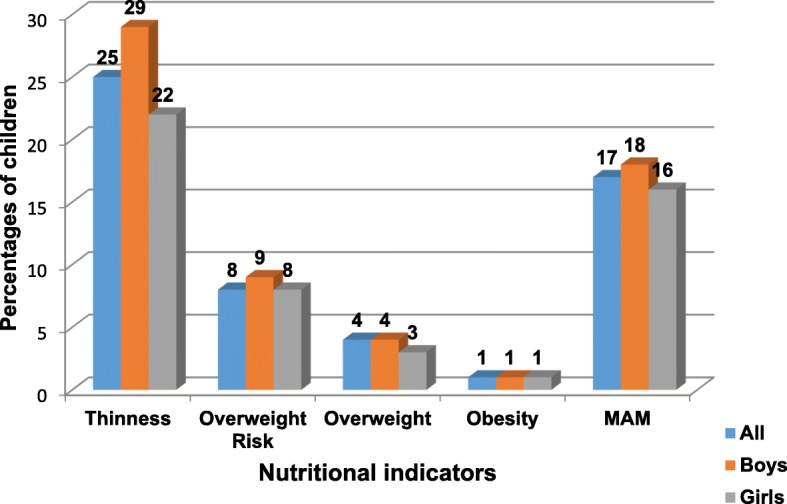
Prevalence of thinness, overweight, obesity, and MAM among children by sex (*n* = 508)

Figure 1 presents the prevalence of thinness, overweight and obesity, and MAM among children. The prevalence of thinness was 25% while for overweight it was 4% and for obesity it was 1%. Twenty-nine percent (29%) of the boys were thin, as were 22% of the girls. For boys and girls respectively, the underweight risk was 9% vs. 8%, the overweight risk was 4% vs. 3%, and the obesity risk was 1% vs. 1%. No significant prevalence of nutritional indicators by BMIZ was observed by sex (*p* = 0.34). The prevalence of MAM was 17% for all children, 18% for boys and 16% for girls, with no significant difference (*p* = 0.58).

**Fig. 2:**
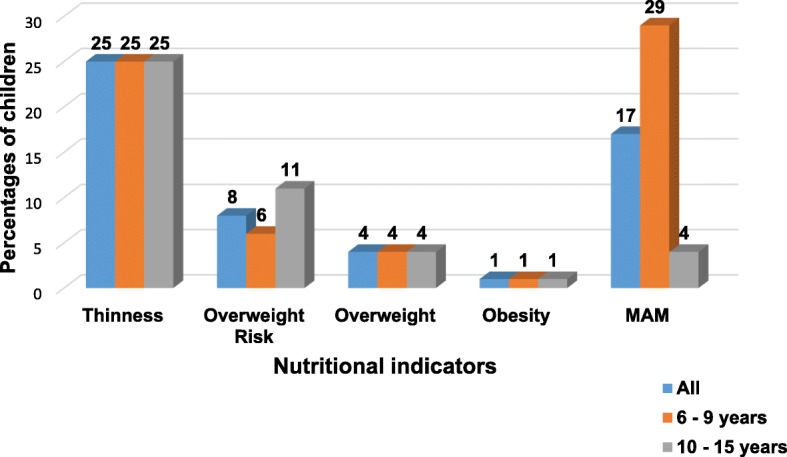
Prevalence of thinness, overweight, obesity, and MAM among children by age groups (n = 508)

In Fig. 2 the prevalence of thinness, overweight and obesity among children are shown with reference to the two age groups; 6–9 years (younger children) and 10–15 years (older children), with no significant difference emerging (*p* = 0.92). The results show a similar prevalence for thinness (25%), overweight (4%) and obesity (1%) for each age group, while the overweight risk was higher (11%) among older children (10-15 years) and lower (6%) in the younger group. The prevalence of MAM was higher for children aged 6–9 years (29%) and very low for older children (4%), with a significant difference (*p* ≤ 0.0001).

### Age and anthropometry of mothers

The age and the anthropometry of mothers are presented in Table 3**.** Slightly, over half of mothers [285; 56%] who participated in this study were aged 35 years and above, while 223 (44%) were below 35 years of age. One hundred and thirty nine [139; (27.4%)] of the mothers were overweight and 215 (42.3%) were obese. Abdominal obesity by waist circumference was observed among 269(52.9%) mothers, while by waist-hip ratio, it was present in 168 (33.1%) mothers.

**Table 3:** Age and anthropometry of mothers (*n* = 508)

Variables	Categories	Frequency	Percentages
Age (years)	< 35	223	44
	≥ 35	285	56
BMI (kg/m^2^)	Normal	142	27.9
	Underweight	12	2.4
	Overweight	139	27.4
	Obesity	215	42.3
Waist Circumference(cm)	Normal	239	47.1
	Abdominal obesity	269	52.9
Maternal waist hip ratio	Normal	340	66.9
	Abdominal obesity	168	33.1

### Sociodemographic status of mothers

Most of the mothers [321; 63.2%] were single, had completed grade 12 (45%), were unemployed with no monthly income [418; 82.3%] and were living on child social grants [441; 86.8%] as their source of income. One-hundred and seventy 170 (33.5%) of the mothers were heads of household. The houses where children lived with their mothers were larger households with 5–9 family members [293; 57.7%)]. Most of the houses were made of bricks [319; 63.2%] and 399 (78.5%) had electricity as a source of energy for cooking and refrigerator use was reported by 426 (83.9%) mothers. Water was accessible in 375 (73.8%) households and the use of pit toilets was reported by 486 (95.7%) mothers (Table 4).

**Table 4:** Socio-demographic characteristics of mothers (*n* = 508)

Variables	Categories	Frequency	Percentages
Mother’s marital status	Ever married	187	36.8
	Single	321	63.2
Mothers’ level of Education	Low literacy	206	40.6
	High literacy	302	59.4
Mother’s employment	Employed	90	17.7
	Unemployed	418	82.3
Mother’s occupation	None	418	82.3
	Civil servant	16	3.1
	Labour/domestic work	74	14.6
Child support grant	Yes	441	86.8
	No	67	13.2
Type of house	Brick	319	63. 2
	RDP/mud/shack	189	36.8
Household head	Self	170	33.5
	Spouse/partner	193	38.0
	Parents	117	23.0
	Grandparents	19	3.7
	Relatives	9	1.8
Household income	≤$70,53	180	35.4
	$70.59 –$352.62	261	51.4
	$352.69 – $705.24	67	13.2
Number of household members	1 to 4	183	36.0
	5 to 9	293	57.7
	≥10	32	6.3
Number of household children	1	30	7.5
	2	132	24.4
	≥3	346	68.1
Source of energy for cooking	Electricity	399	78.5
	Firewood	83	16.3
	Paraffin	17	3.4
	Gas	5	1.0
	Coal	4	0.8
Refrigerator use	Yes	426	83.9
	No	82	16.1
Access to water	Yes	375	73.8
	No	133	26.2
Type of toilet	Flush toilet Pit toilet	22 486	4.3 95.7

### Determinants of thinness and overweight/obesity

The results of a logistic regression analysis to determine the association of thinness and overweight/obesity with several factors are presented in Table 5. After controlling for all independent variables, there were positive associations between thinness and child’ sex and maternal BMI, and a negative association with access to water. Boys were more likely to be thin than girls [AOR = 1.54, 95%CI =1.01–2.35, *p* = 0.05]. Mothers who were overweight/obese were more likely to have thin children [AOR = 1.48, 95%CI = 1.01–2.18, p = 0.05] and children who lived in households that had water access were less likely to suffer from thinness [AOR = 0.58, 95%CI = 0.34–0.97 *p* = 0.04]. On the other hand, there were negative associations between overweight/obesity and learning grade and maternal BMI. Children who were in the lower learning grades (grade 1 to grade 4) were less likely to be overweight/obese [AOR = 0.48, 95%CI = 0.01–0.84, *p* = 0.01] than children in learning grades 5 to 7. Mothers who were overweight/obese were less likely to have overweight/obese children [AOR = 0.19, 95%CI = 0.07–0.45, *p* ≤ 0.0001].

**Table 5:** Multivariate analysis of determinants for thinness and overweight/obesity among schoolchildren

Thinness	AOR	95%CI	P-value
Child’ sex	1.53	1.01–2.35	0.05*
Maternal BMI	1.48	1.01–2.18	0.05*
Maternal WHR	1.41	0.89–2.21	0.14
Access to water	0.58	0.34–0.97	0.04*
Overweight/obesity			
Learning grade	0.48	0.28–0.84	0.01*
Maternal BMI	0.18	0.07–0.46	≤0.0001*
Maternal marital status	0.63	0.36–1.10	0.10
Maternal education	1.65	0.94–2.88	0.08

* indicates significance

## Discussion

The paper studied the double burden of malnutrition and determined the prevalence and determinants of thinness and overweight/obesity using child-mother paper in a rural DHDSSS, South Africa. The study population was characterised by a depressed socioeconomic status indicated by high rates of unemployment, no income, dependence on child support grant, as well as, unfavourable circumstance such as singlehood and low rates of tertiary education. In addition, the poor infrastructure in terms of sanitation and the dwelling environment was also observed. These living conditions are persistently similar to the reports of studies conducted in DHDSSS [10, 11, 27] and almost similar to some of the findings of the South African National Health and Nutrition Examination Survey (SANHANES-1) and the South Africa Demographic and Health Survey (SADHS), 2016 [24, 38].

Furthermore, our study, reported a high prevalence of undernutrition in a form of thinness (25%) among the child participants, affecting 29% of boys and 22% of girls. Mild acute malnutrition was 17%, particularly, high among children aged 6 to 9 years (29%). This is despite these children being provided with meals at the schools through the NSNP. Some of the reported challenges with the NSNP are children not receiving the recommended amount and type of foods, and the nutritional quality of meals being less than optimal, [39]. The HAKSA, in their report, indicated that the NSNP is only succeeding to provide optimal nutritious meal for half of children and youth [22].

The high prevalence of thinness among the child participants in this study is in contrast with other rural South African settings, which have reported lower low prevalence of thinness at 1% [9] and 6% [3] among the children in a rural area. The SANHANES-1, the South African Vitamin A Consultative group (SAVACG, 1995) and the National Food Consumption Survey (NFCS, 1999; 2005), reported low prevalence of thinness among children at 2.6, 3% and 4–5%, respectively [24, 40, 41]. However, compared to studies conducted in other countries, the prevalence of thinness among schoolchildren in this study is lower than the average prevalence of thinness in both Africa and South East Asia (35%) [42]. For example, countries such as Ethiopia (37%), India (76%) and Bangladesh (32%) report a higher prevalence rate of thinness among schoolchildren [43–45]. It is worth noting that the prevalence of thinness among schoolchildren in this study is higher than the global prevalence estimated at 10 to 15% and has barely changed over the past decade [46].

The variations in the prevalence of thinness among children in South African studies suggest that topography might play a role [47]. In addition, poverty and food insecurity, which are more severe in rural settings, have been implicated [41, 48]. Similarly, we attribute thinness among schoolchildren to the poor socio-economic status present in the households these children live in. This circumstance suggest that the ability to purchase nutritious food is compromised, which may lead to food unavailability in households and ultimately affect the feeding practices of children, as explained by various researchers [49, 50]. The variations in the prevalence of thinness across and within different countries is explained by differences in their social, demographic, economic, nutritional and cultural contexts [51–53].

In addition to persistent undernutrition among schoolchildren, a high prevalence of overweight (27.4%) and obesity (42.3%) was reported among their mothers in this study. High prevalence of overweight and obesity (55%) with 27 and 28% among women of childbearing age, respectively, with micronutrients deficiencies, have been reported in the same rural region, a decade ago [10, 54]. Recent reports showed that the prevalence of overweight/obesity (54 to 76%) [11, 13] is on the rise in the same region where stunting among children has been estimated at 48% [9]. In a period of 1998 to 2016, the SADHS reported an increase the prevalence of overweight/obesity from 56 to 68%. Currently, 27% of women of reproductive age are overweight while 41% are obese [38]. The SADHS further estimated abdominal obesity by waist circumference ranging from 41 to 45% (1998–2016) [38], almost similar to 52.9%% reported in our study.

The findings of this study, characterise the double burden of malnutrition, similar to other studies conducted in various rural settings, that have reported the double burden of malnutrition, mainly on an individual community/region level in South Africa [3, 9]. A prospective cohort study conducted among children aged ≤3 years in rural villages amidst transition, reported that 48% of children (≤3 years) were stunted, 18% were overweight, 24% were obese, and 19% were stunted and overweight or obese [9]. Another cross-sectional growth survey conducted among children and adolescents (aged 1–20 years), reported the co-existence of substantial levels of undernutrition, particularly stunting at an early age, with marked levels of overweight/obesity in adolescent girls in the same socio-geographic transitional population [3].

The coexistence of child undernutrition and maternal over nutrition in the same households and even the same community as in this study, could be due to the consumption of energy-dense food that leads to overweight/obesity among the mothers, while the energy dense foods are not sufficiently nutrient dense to provide the children with adequate nutrition, leading to undernutrition [55]. Although, previously, the double burden of malnutrition was more common in households with higher income [56], the current study shows that the double burden of malnutrition is common in households with a depressed socio-economic status characterised by a high rate of unemployment and a low household monthly income.

On the other hand, our findings showed a low prevalence of overweight (4%) and obesity (1%) among the child participants. The rate of prevalence of obesity and overweight is almost similar to that of overweight (5%) and obesity (1%) among children in a rural setting [47]. The prevalence of overweight (9%) and obesity (3.8%) have been reported in other rural settings in South Africa [57]. The prevalence of overweight and obesity were equally low for boys (4.3 and 0.5%) and girls (3.3 and 1.3%), and for 6–9 year olds (3.9 and 1.2%) and of 10–15 year olds (3.5 and 0.8%), respectively. Nevertheless, 11% of children aged 10-15 years in this study were at the risk of developing overweight.

In the past two decades, the NFCS (1999; 2005) reported the national prevalence of overweight and obesity among South African children (1–9 years) at 10 to 12% and 4 to 5%, respectively [41]. However, the SANHANES-1 estimated the combined prevalence of overweight and obesity for South African children aged 6–14 years at 14.2% [24], which is higher than the prevalence in the current study. The Youth Risk Behaviour Survey (YRBS, 2002) reported a 17% prevalence of overweight and 4% of obesity among school going youth aged 13 to 19 years [58]. The surveys reported that boys were more affected by thinness and undernutrition was most prevalent in the rural area [24, 41, 58]. Among school-aged children, the prevalence of overweight and obesity in LMICs vary from 5.7% to over 40%, while globally, the prevalence is estimated at 10% [59].

In the current study, the low prevalence of overweight and obesity among children may be due to household poverty, still, as indicated by the poor socioeconomic status found. Also, walking has been reported as the most common mode of travel used by learners (82.5%) in Limpopo Province to get to school [60]. That might also have diminished overweight and obesity among the children in this study. However, it is worth noting that the prevalence of overweight and obesity among children in other countries such as Mozambique (1, 0%), Togo (5.2, 1.9%) and Senegal (1, 0%) [61–63] were almost similar to our findings (4, 1%).

Socio-demographic variables and maternal age and anthropometry as the risk factors for thinness and overweight/obesity among children have been studied and implicated in several studies [53, 64]. The current study did not establish a causal relationship between thinness and overweight/obesity and covariates, but the independent strength of association was determined. The study found that boys had a slightly higher prevalence of thinness than girls (29% versus 22%) did, although the difference was not significant. However, logistic regression analysis showed that the boys were more likely to be thin than the girls [AOR = 1.53 (CI: 1.01–2.35), *p* = 0.05]. Our finding of a significant association between child’ sex and thinness is similar to that in a study in Ethiopia [20]. Research has suggested that inadequate nutrient intake and higher physical activity levels among boys might be the reason while girls are less likely to be thin than boys [65, 66]. Furthermore, children in lower learning grades (1 to 4) were less likely to be overweight/obese compared to children in learning grades; 5, 6 and 7 [AOR = 0.48 (CI: 0.28–0.84), *p* = 0.01]. The lesser likelihood of children in lower learning grades (grades 1 to 4) to be overweight/obese found in this study is consistent with the findings in other studies [67, 68]. Almost certainly, the vicious cycle of poverty predisposes to a chronic undernutrition from an early age but acute undernutrition might be the cause in this study.

The study also showed a significant association between maternal BMI with thinness [AOR = 1.48 (CI: 1.01–2.18), *p* = 0.05] and overweight/obesity [AOR = 0.18 (CI: 0.07–0.46), *p* ≤ 0.0001]. The association of overweight/obese mothers and thin children is similar to the association found in studies in Chile and India [69, 70]. Researchers argue that in areas where there is food insecurity and a high level of poverty, the mothers access lower quantities of food with high energy levels, which may lead to child thinness since they are not nutrient dense, while among mothers, overweight/obesity may result [71]. In addition, children who lived on households with access to water were found to be less likely to suffer from thinness than those who had no access [AOR = 0.58 (CI: 0.34–0.97), *p* = 0.04]. This result is consistent with those of other studies [72, 73]. Access to an improved water source is found to be an indicator of the higher probability of safe water [74]. The literature documents that the contamination of drinking water may particularly jeopardise children’s nutritional status by inhibiting their growth and health, leading to malnutrition [72].

One of the limitations of this study was that we could not establish the cause-effect relationship between the outcome variable and the covariates because of the study design. Longitudinal studies, which follow their participants and study the causes and trends of thinness among schoolchildren in deep rural areas, are necessary. However, since there is supporting evidence, the effect estimated in this study could be a good measure of the association between the identified factors and thinness and overweight/obesity. Another limitation could be that we did not obtain information on individual and household dietary intake, which could have given us valuable information in the study area and explain the double burden of malnutrition.

## Conclusion

The study contributes critical foundational data in describing the persistence of acute malnutrition among schoolchildren in DHDSSS. Thinness was more prevalent among the child participants in this site, and it is a clearer public health problem than childhood overweight/obesity. On the other hand, a higher prevalence of overweight and obesity among the participants’ mothers were observed. This study confirms the presence of a double burden of malnutrition on household and community levels in this area. The socio-demographic variables of the mothers suggested household poverty. In almost all of the households in which the children were being raised, the mothers were unemployed, were single rather than married, depended on the monthly child social grant, and had no other monthly household incomes.

The determinants of thinness and overweight/obesity in this study were child’ sex, learning grade, maternal BMI, and access to water. This study supports a need to address the dual problems of undernutrition and the rapidly rising trends of overweight and obesity in South Africa. Using a holistic approach from the context point of view, in addition to the possible risk factors, to improve the nutritional status of schoolchildren in DHDSSS is imperative. Moreover, we are in agreement with SANHANES-1, that investing in nutrition of the population will lead to long-term benefits for South Africa [24]. Hence, emphasis should be given to involving nutritionists and educating the mothers about self and child nutritional care.

## Data Availability

The dataset for schoolchildren and their mothers generated and analysed during the current study are available from the corresponding author upon reasonable request.

## Abbreviations

DHDSSS: Dikgale Health and Demographic Surveillance System Site
HAKSA: Healthy Active Kids South Africa
INDEPTH: International Network for the Demographic Evaluation of Populations and their Health
NDoH: National Department of Health
NFCS: National Food Consumption Survey
NSNP: National School Nutrition Programme
SADHS: South Africa Demographic Health Survey
SANHANES-1: South African National Health and Nutrition Examination Survey
SAVACG: South Africa Vitamin A Consultative Group
